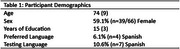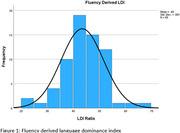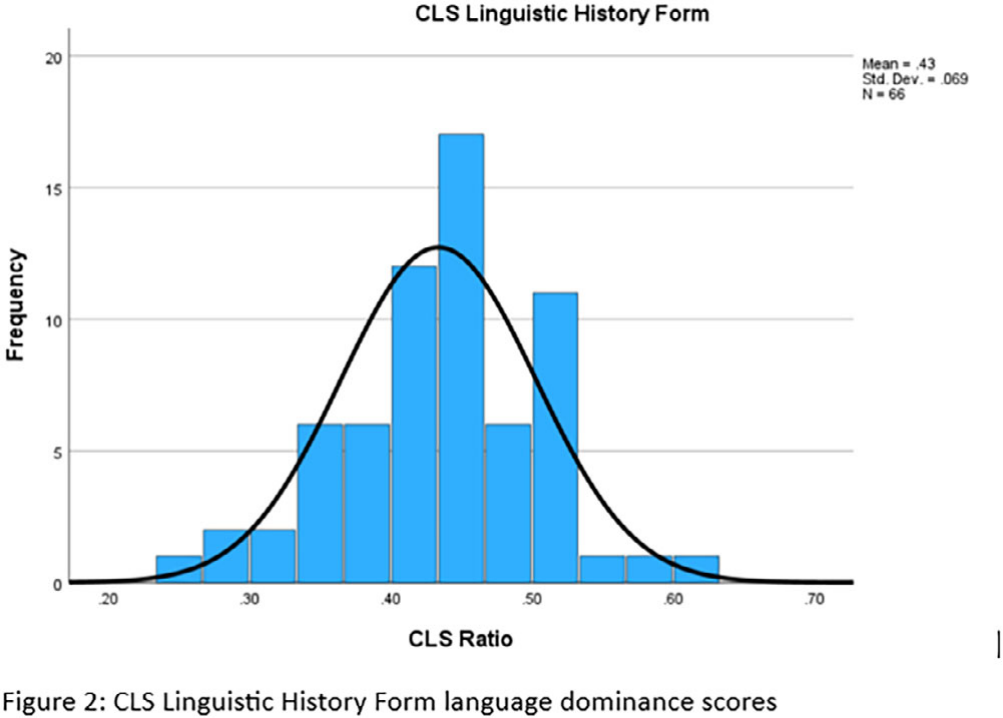# Determining Language of Neuropsychological Assessment in a Bilingual Population

**DOI:** 10.1002/alz70857_105625

**Published:** 2025-12-25

**Authors:** Gabrielle Hromas, Stephanie Santiago‐Mejias, Chen‐Pin Wang, Roman A. Fernandez, Gladys E. Maestre, Silvia Mejia‐Arango, Jennifer G Del Bosque, Roberto Garcia, Denisse Cisneros Garcia, Samantha Gates, Patricia Hernandez, Marialy Salinas Valdez, Amaya M Seidl, Hector A Trevino, Juan Toranzo, Marucela Uscamayta Ayvar, Angel G Velarde, Vanessa M. Young, Jessica Zapata, Katya Rascovsky, David A. Gonzalez, Sudha Seshadri, Ashley LaRoche, A. Campbell Sullivan

**Affiliations:** ^1^ Glenn Biggs Institute for Alzheimer's & Neurodegenerative Diseases, University of Texas Health Science Center, San Antonio, TX, USA; ^2^ Glenn Biggs Institute for Alzheimer's and Neurodegenerative Diseases, University of Texas Health Science Center, San Antonio, TX, USA; ^3^ South Texas Alzheimer's Disease Research Center, Harlingen, TX, USA; ^4^ The University of Texas Rio Grande Valley School of Medicine, Harlingen, TX, USA; ^5^ RGV Alzheimer's Resource Center (AD‐RCMAR), Harlingen, TX, USA; ^6^ Penn FTD Center, Perelman School of Medicine, University of Pennsylvania, Philadelphia, PA, USA; ^7^ University of Pennsylvania, Philadelphia, PA, USA; ^8^ Rush University Medical Center, Chicago, IL, USA

## Abstract

**Background:**

Determining the language of neuropsychological assessment in a bilingual patient population is difficult with implications for test performance and clinical interpretation. We compared two methods of identifying language dominance for neuropsychological testing in a sample of bilingual (English/Spanish) Hispanic/Latine patients in South Texas.

**Method:**

Research participants from the South Texas Alzheimer's Disease Research Center (STAC), who identified as bilingual were administered the CLS: Linguistic History Form, which asks individuals to provide a self‐rating of their English and Spanish language proficiency, and verbal fluency tasks (F/L fluency in English, and P/M fluency in Spanish) to create a language dominance index (LDI) based on objective test performance.

**Result:**

*N* = 66 participants completed both measures of language proficiency at the time of data extraction (Table 1). Ratios were calculated for each measure to determine a standardized language proficiency score. Scores ≤ 0.49 were classified as Spanish‐dominant, scores = 0.5 were classified as purely bilingual, and scores ≥ 0.51 were classified as English‐dominant (Figures 1 and 2). Paired samples t‐test comparing the language proficiency ratios revealed that both classification methods performed similarly (*t*(65)=0.175, *p* = .431). With the CLS, 5 participants were classified as Spanish‐dominant, 9 as purely bilingual, and 52 as English‐dominant. With the verbal fluency‐derived LDI, 9 individuals were classified as Spanish‐dominant, 4 as purely bilingual, and 53 as English‐dominant.

**Conclusion:**

Here, we saw subtle (non‐statistically significant) differences in subjective rating of language proficiency and objective measurement of language dominance. CLS classified 13.6% and 7.6% of the sample as bilingual or Spanish‐speaking, respectively, while LDI classified 6.1% and 13.6% as bilingual or Spanish‐speaking. A high percentage of the sample was classified as English‐dominant regardless of measure (80.3% and 78.8%), possibly due to demographic factors (e.g., recruitment site, education level, and country of origin). Future analyses will explore these additional demographic influences as well as cognitive testing outcomes in a larger sample across multiple sites in South Texas.